# Voluntary Attention Assessing Tests in Children with Neurodevelopmental Disorders Using Eye Tracking

**DOI:** 10.3390/children11111333

**Published:** 2024-10-31

**Authors:** Anna Rebreikina, Dmitry Zakharchenko, Antonina Shaposhnikova, Nikita Korotkov, Yuri Klimov, Tatyana Batysheva

**Affiliations:** 1 Laboratory of Human Higher Nervous Activity, Institute of Higher Nervous Activity and Neurophysiology of RAS, 117485 Moscow, Russia; 2 Scientific and Practical Center for Pediatric Psychoneurology of the Moscow Department of Health, 119602 Moscow, Russia

**Keywords:** eye tracking, video oculography, attention assessment, voluntary attention, neurodevelopmental disorders

## Abstract

**Background/Objectives:** The development of techniques for assessing cognitive functions using eye tracking is particularly important for children with developmental disabilities. In this paper, we present pilot results from the validation of two methods for assessing voluntary attention based on eye tracking. **Methods:** The study involved 80 children aged 3 to 8 years with neurodevelopmental disorders. Children performed two eye-tracking tests in which they had to ‘catch’ a stimulus by looking at it. They also completed the Attention Sustained subtest of the Leiter-3 International Performance Scale. In the first test, the stimuli were presented at different locations on the screen in subtests with stimuli onset asynchrony of 2 s and 1 s. A translucent blue marker marked the position of the gaze on the screen. The number of trials in which the gaze marker approached the stimulus was determined. In the second test, the location of the stimuli on the screen was changed based on gaze fixation in the ROI area. The time taken to complete the task was evaluated. **Results:** The results of both eye-tracking tests showed significant correlations with scores on the Attention Sustained Leiter-3 subtest and significant test–retest reliability. **Conclusions:** The results indicate that the present eye-tracking tests can be used for assessing voluntary attention in children with some neurodevelopmental disorders, and further research is warranted to assess the feasibility of these tests for a broader range of developmental disorders. Our findings could have practical implications for the early intervention and ongoing monitoring of attention-related issues.

## 1. Introduction

Attention plays a significant role in a child’s development, as it shapes how they perceive and interact with the external world. It is common to distinguish between voluntary and involuntary attention. Involuntary attention is labeled as “exogenous” [1], bottom–up, transient, and stimulus-driven [2]. Voluntary attention, also called endogenous, top–down, and sustained, refers to the conscious act of directing attention to a specific activity or situation, and, as such, involves a certain level of effort or intentionality [1,2,3,4,5]. Voluntary attention is essential for learning and social interaction, allowing children to organize and control their behavior, concentrate on tasks for long periods of time, and ignore distractions. Thus, assessment of voluntary attention is important for the timely identification of childhood developmental problems, monitoring attentional development during child maturation, and evaluating the effectiveness of rehabilitation interventions.

It is important to note that there are limited tests available for practical use in assessing attention in children aged 3–8 years and, of the tests available, not all are adapted for different languages [6,7,8]. Many of the published assessment methods remain experimental and inaccessible to practitioners. A detailed list of attention assessment tests was presented in Mahone and Schneider, 2012 [6]. This list can be also supplemented with the Test of Everyday Attention (TEA-Ch, for children from 6 years old), Early Childhood Attention Battery (ECAB) for children 3–6 years old [7,8], attention subtests of the Leiter International Performance Scale, Third Edition (Leiter-3, for ages 3 years and older) [9]. All of these tests require specific motor or verbal responses, specific skills (for example, knowledge of numbers), or are designed for children over the age of five. Some of the techniques take a long time to complete, making them difficult to use even with healthy 3–4-year-olds and even more so with children with neurodevelopmental disorders.

This highlights the need for new approaches to the assessment of attention in children with neurodevelopmental disorders, especially those with motor impairments. Among the current literature are promising methods of attention diagnostics based on eye tracking (or video oculography). Many studies prove that the direction of gaze reflects the focus of a person’s attention. Evaluation of various measures of eye movement can provide a non-invasive indicator of brain function and cognitive abilities [10]. Several papers have shown the value of eye tracking in assessing the effectiveness of rehabilitation interventions where traditional attention tests are ineffective or not possible due neurodevelopment problems [11,12,13]. Despite the rather large number of existing eye-tracking studies investigating both neurotypical children and children with various neurodevelopmental disorders, there are limited studies devoted to the development of diagnostic eye-tracking techniques for practical application (see Rebreikina and Liaukovich, 2024 for a review [14]).

Of those in devising, there is the Utrecht Tasks for Attention in Toddlers Using Eye Tracking (UTATE) for assessing 12–25-month-old children [15,16,17,18]. Several attention tasks are included in the Eye-Tracker Intelligence Screening Test for Children with Generalized Developmental Delay (for 1.5–4-year-old children) [19], but this test does not provide a separate measure of attention. Eye-tracking techniques are also being developed to diagnose ADHD in schoolchildren [20,21]. It should be noted that most of the oculographic parameters used in these methods require good quality data (calibration and validation), which are often difficult to obtain in children with developmental disorders. Their analysis is rather labor-intensive and requires special training of assessors. The approaches proposed in the UTATE are mainly designed for assessing involuntary attention in infants. Approaches for diagnosing ADHD, on the other hand, are too complex for children of preschool age with developmental disorders and older children with more severe developmental disabilities than ADHD. Due to the above-mentioned problems, there is a need for a simple and universal test based on eye tracking to assess voluntary attention in children with developmental disorders.

In this paper, we present the results of a pilot study of two tests we developed for assessing voluntary attention using eye tracking in children with developmental disorders. The tests present many advantages over existing methods, both for children and assessors. For children, testing only takes around 5 min, and the tasks and instructions are easy and do not require any prior knowledge or skills. For assessors, the tests do not require high-accuracy eye-tracker calibration, the simple metrics can be evaluated even by non-specialists, and the results provide qualitative and quantitative indicators that can be used to assess a child’s attentional development in cases where standard methods of assessment are not possible.

The aim of the study was to show the possibility of using the two tests on children with developmental disorders to evaluate the convergent validity and reliability of the obtained data and identify weaknesses and limitations in order to further improve the tasks.

## 2. Materials and Methods

### 2.1. Participants

Eighty children aged 3.5–8.9 years (5.67 ± 1.3, 55 males) participated in the study: 30 children (5.91 ± 1.21, aged from 4 to 7.8, 25 males) participated only in the validation of the first test. A second test was later added to the experimental procedure, and a further 50 children (5.54 ± 1.34, aged from 3.5 to 8.9, 30 males) participated in the validation of both the first and second tests. Children with various neurodevelopmental disorders potentially capable of performing the pen-and-paper Attention Sustained subtest of the Leiter International Performance Scale, Third Edition (Leiter-3) were invited to participate in the present study. All were able to use a felt-tip pen, had normal or corrected-to-normal vision and did not have motor disorders. The diagnoses of the children according to the 10th revision of the International Classification of Diseases (ICD-10) were: F84.8—Other pervasive developmental disorders (PDD); F80.1—Expressive language disorder (ELD); F84.0—Autistic disorder (AD); F70.08—Mild mental retardation with the statement of no, or minimal, impairment of behavior; F90.1—Attention deficit hyperactivity disorder (ADHD); R47.8—Other and unspecified speech disturbances; G80.1—Spastic diplegic cerebral palsy; G93.4—Encephalopathy, unspecified; and G96.8—Other disorders of the nervous system.

Distribution of the 80 children by age, sex, and diagnosis is presented in Table 1. The children were recruited during a rehabilitation course at the Scientific and Practical Center for Pediatric Psychoneurology of the Moscow Department of Health. The study procedure was approved by the Ethical Committee of the Institute of Higher Nervous Activity and Neurophysiology of RAS, 117485, Moscow, Russia, protocol #4 dated 8 June 2023. All aspects of the research conformed to the tenets of the Declaration of Helsinki. Parents or legal representatives gave written consent for the children’s participation in the study.

Since the aim of the study was primarily to investigate the possibility of assessing voluntary attention using our tests in children with developmental disorders, rather than the specific features of attention in different clinical groups, we did not divide the children into groups depending on the diagnosis and did not include a control group. Five children with more severe disorders were unable to complete any of the tasks because they had difficulty understanding speech, refused to sit or would not look at the screen. In addition, three children did not complete the Attention Sustained subtest. The analysis of the first test therefore included data from 72 children, while the analysis of the second test included data from 44 children. All children included in the analysis of the second test also performed the first test.

### 2.2. Tests Description

Both tests were developed in C# and carried out using the Gazepoint GP3 eye tracker (Gazepoint, Vancouver, Canada) with a sampling rate of 60 Hz, 0.5–1 degree of visual angle accuracy. The eye tracker allowed participants to move 25 cm horizontally, 11 cm vertically and ±15 cm closer and further away from the screen without losing track of the gaze. Completion of tasks was recorded using video recording of the computer screen.

#### 2.2.1. The White Dots Test

**Figure 1:**
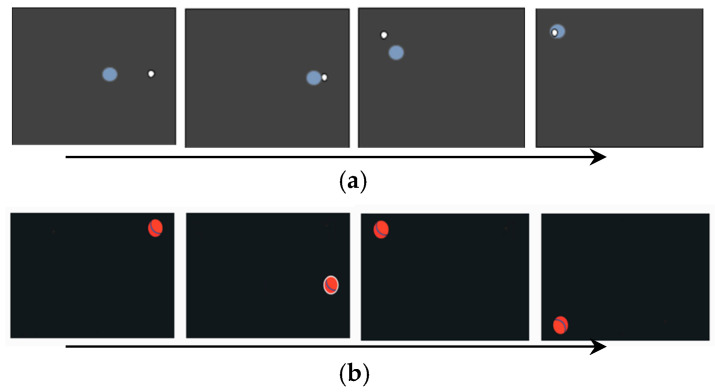
Design of: the White Dots test (**a**); the Red Balls test (**b**).

The first test, White Dots, consisted of 4 subtests in which 30 stimuli were presented on a dark gray background at different pseudorandom positions on the computer screen. The stimuli were white dots with a diameter of 0.5 degrees of visual angle (Figure 1a). Stimuli were presented in a pseudorandom order in different parts of the screen, and the distance between stimulus presentation locations ranged from 15 to 30 degrees of visual angle. In two subtests, stimuli were presented with a stimulus onset asynchrony (SOA) of 2 s, and in the other two subtests, stimuli were presented with an SOA of one second. These presentation speeds were chosen based on the results of a preliminary validation of the test which showed that the task with an SOA of 1 s was more difficult. The presentation order of the subtests was as follows: subtest 1 (SOA 2 s), subtest 2 (SOA 1 s), subtest 3 (SOA 1 s), and subtest 4 (SOA 2 s). The child’s task was to “catch” all of the white dots with a translucent blue marker (diameter 2.5 degrees of visual angle) marking the position of the child’s gaze on the screen. Before testing began, the child was shown that the blue marker moved to where the child was looking. The instructions for the child were as follows: “Now you are going to play catch, you will have to catch a small white ball that will appear on the screen and run away from you. To catch the ball you need to look at it as quickly as possible. The white ball is caught when the blue circle comes close to it”.

When the child looked at (“caught”) the white ball, the child was encouraged by the assessor with the words: “Well done, you caught it”! If the child stopped completing the task (missed 3 targets in a row), he/she was reminded of the need to look at the white ball. If the child, despite this, did not resume completion of the task, then the researcher directed his/her attention to the stimuli using a pointing gesture until the child looked at two consecutive stimuli that the researcher showed. If the child stopped performing the task independently again, the experimenter continued to help with pointing gestures until the end of the subtest, as described above. Assessment of the task performance was carried out using the video recording based on which the number of trials a child completed by him/herself (without any help from an adult) in each subtest was determined. Additionally, the number of verbal reminders and uses of pointing gestures were assessed.

#### 2.2.2. The Red Balls Test

The second test, Red Balls, was developed after studies with the first test had already begun, since some children with severe developmental delays experienced difficulty completing White Dots. We hypothesized that assessing the attention of such children is possible using a shorter task with more colorful and larger stimuli. The task worked using interactive feedback from gaze fixation on the area of interest. The on-screen gaze marker was not used in this test unlike the White Dots test. In this task, children were presented with three sequences of 11 red balls (diameter 31.8 mm, 3.03 degrees of visual angle on a dark gray computer screen) which, as in the first task, had to be “caught” by looking at them (Figure 1b). When the child looked at the ball, a white ring appeared around it—a “trap”. The ball was “caught” if the child fixed his gaze for 300 ms in the region of interest (ROI), which was exactly the size and location of the ball, after which the ball disappeared and the next stimulus was immediately presented in another place on the screen (the distance between the presentation sites was from 15 to 30 degrees of visual angle). The subtest ended after fixing the gaze on 11 balls. The time required to complete each of the three subtests was assessed. If the child was distracted from completing the task (did not look at the ball for 3 s or longer after it appeared), he/she was verbally reminded of the need to catch the balls. If the reminder did not help, then attention was drawn to the balls using a pointing gesture from the assessor, as described in the White Dots tests. The time to complete the test increased even in cases of assessor assistance. Thus, the test completion time was a summary indicator of voluntary attention and the ability to complete tasks independently.

### 2.3. Procedure

Children were positioned with a distance of about 60–65 cm from the eyes to the center of the computer monitor (17-inch diagonal, 1280 × 980 dpi resolution). Children sat in the chair on their own or were held in their parent’s arms. In the former case, the parent sat slightly behind and to the left of the child. The experimenter was to the right of the child during the experiment and, if necessary, the experimenter or the parent corrected the child’s position on the chair, gently holding the back of the child’s head to reduce head movements. The chin rest was not used. The camera was to the side of the participants capturing the screen and hand movements in front of the screen and audio recording the conversation. Calibration of the eye-tracker was carried out using 5 points. Children then completed the eye-tracking tasks.

To assess the validity of the task, children performed the Attention Sustained subtest of the Leiter International Performance Scale, Third Edition (Leiter-3) on paper. This test assesses sustained visual attention and requires visual scanning skills as well as the ability to inhibit repetitive motor responses when crossing out stimuli [9]. In accordance with their age, the child performed 2–4 tasks of the Attention Sustained subtest. The child’s task was to mark the given images (or pattern of images) with a felt-tip pen on paper within a limited time (30 s or 60 s, depending on the task and age). The number of marked images and errors was counted, and a scaled score of task performance was determined according to the child’s age. The Attention Sustained subtest was chosen for control testing because it is standardized for different age groups, quick and simple enough to complete, and is primarily aimed at testing sustained attention, i.e., the ability to concentrate on a monotonous task, which is closest to our eye-tracker paradigm.

Children performed the Attention Sustained subtest immediately after eye-tracking testing or the next day in case of exhaustion of the child.

To determine whether the test was reliable, some children were retested (the interval between tests was 1–10 days, N = 34 for the White Dots tests and N = 27 for Red Balls tests).

### 2.4. Data Analysis

In the White Dots test, not all children were able to complete all four subtests: some children did not want to continue the task due to loss of interest in it, and in this case, the experiment was stopped. Only 55 children completed all four subtests. As a consequence, quantitative analyses were conducted for subtest 1 (SOA 2 s, N = 72) and for the subtest 2 (SOA 1 s, N = 63). The tests were analyzed in two ways:

1. Visual analysis: The number of trials in which a child looked at the target independently was determined by visually analyzing the videos. The trial was determined to be complete if a child looked at the target (i.e., the blue gaze marker came within an area of about 4 cm radius around the stimulus) without any repetition of instructions or guidance. If a child looked at the stimulus after it disappeared, that was also counted as a successful trial. The gaze to the first stimuli was not taken into account.
2. Automatic analysis: Using a specially written MATLAB code (MATLAB 2018b, The MathWorks, Inc., Natick, MA, USA), the number of trials in which the child’s gaze fell within a region of interest (150 pixels, or 3.79 angular degrees) around the stimulus was determined (except for the first stimuli). The ROI was determined on the basis of a preliminary analysis of eye movement graphs; it did not overlap with the zones of interest of two consecutive stimuli. If the gaze did not fall within the ROI, the trial was considered a miss. The number of trials considered to have been performed successfully was higher in the automatic analysis, as the code used did not account for any verbal or behavioral assistance given by the assessor.

For the Red Balls test, the time taken to complete each subtest was calculated from the appearance of the first stimulus to the disappearance of the last stimulus. The average time taken to complete the three subtests was also determined.

To assess convergent validity, we used correlation analyses (Pearson partial correlation analysis, controlling for age) of the scaled score of the Attention Sustained subtest and the number of independently completed trials in the first and second subtests of the White Dots test. We also performed correlation analyses (Pearson partial correlation analysis, controlling for age,) of the scaled score of the Attention Sustained subtest with the mean time to complete the Red Balls test and with the time to complete each subtest. According to the standard [22], correlations between 0.10 and 0.29 were interpreted as a small association, between 0.30 and 0.49 as a medium association, and above 0.50 as a large association [18].

Reliability was determined using Pearson correlation of the first and retest results for the White Dots and the Red Balls tests [18], as split-half reliability assessment [15,17] could not be applied in the present study. In line with previous studies, correlations above 0.70 are regarded as “good reliability” and correlations between 0.50 and 0.70 are regarded as “adequate reliability” [18,23]. The statistical analyses were performed in the software R (R version 4.3.2 (2023-10-31 ucrt), R Core Team, 2020).

Additionally, the mean latency of gaze fixation on the ROI during the first and second subtests of the White Dots test was determined using an automatic algorithm developed for MATLAB. ROI was defined as the area surrounding the stimulus with a diameter of 150 pixels. A correlation analysis (Pearson partial correlations controlling for age) of the mean gaze latency with performance on the Attention Sustained subtest was performed.

## 3. Results

### 3.1. The White Dots Test

Seventy-two children completed the Attention Sustained subtest of the Leiter-3 international productivity scales and at least the first subtest of the White Dots test. The distribution of children according to their performance on the Attention Sustained subtest, as well as the average number of completed trials for each subgroup, is shown in Table 2. These preliminary data suggest that if fewer than 23 trials are completed in subtest 1 (SOA 2 s), or fewer than 18 trials are completed in subtest 2 (SOA 1 s), it is highly likely that the child has problems with voluntary attention. Nine children had very low scores on the Attention Sustained subtest (0 points), but even so, the mean number of trials they completed in the White Dots test was 10.7. This indicates that the White Dots test allows assessment of the level of attentional development, at least in some cases where a child is unable to perform the Attention Sustained subtest of the Leiter-3. Even in instances when the child was not able to independently perform the White Dots test, some children began to perform with the help of an adult, allowing for the assessment of attentional development.

**Figure 2:**
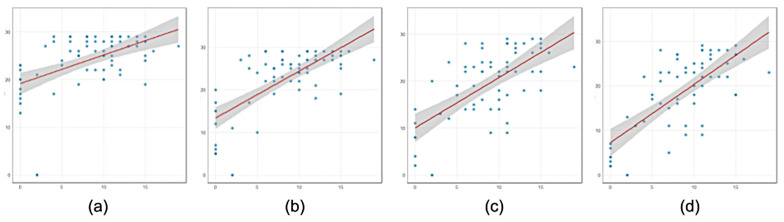
Correlation plots of the number of performed trials (the vertical axis) with the Leiter-3 Attention Sustained subtest score (horizontal axis): (**a**) subtest 1, automatic analysis (SOA of 2 s); (**b**) subtest 1, video analysis; (**c**) subtest 2, automatic analysis (SOA of 1 s); (**d**) subtest 2, video analysis.

Convergent validity analysis showed that the results of the Attention Sustained subtest were significantly positively correlated with the number of completed trials determined by analyzing video recordings of subtest 1 (r = 0.708, *p* < 0.001, N = 72) and subtest 2 (r = 0.746, *p* < 0.001, N = 63). Similar results, but with a lower level of correlation significance, were obtained when the number of trials performed was automatically determined (in subtest 1, r = 0.527, *p* < 0.001 and in subtest 2, r = 0.712, *p* < 0.001) (Figure 2).

Additional correlation analyses of performance on the Attention Sustained subtest with the number of trials completed in subtest 1 (determined by video analysis) also showed significant correlations when the children were grouped into older and younger age brackets. In the subgroup of children 5 years and older, the correlations were r = 0.68, *p* < 0.001 (subtest 1, N = 46) and r = 0.70, *p* < 0.001 (subtest 2, N = 45), and in the 3–4-year-old group, the correlation was r = 0.76, *p* < 0.001 (subtest 1, N = 26) and r = 0.78, *p* < 0.001 (subtest 2, N = 19).

The correlations of the mean latency of gaze fixation on the ROI in the first and second subtests and the Attention Sustained subtest were r = −0.341, *p* = 0.004 (subtest 1) and r = 0.199, *p* = 0.126 (subtest 2), respectively.

Additionally, a comparison of performances on the first and fourth subtests in children who completed all four subtests (N = 55) showed the number of completed samples did not change significantly in 33 people, decreased in 14 children, and increased in 8 children.

### 3.2. The Red Balls Test

**Figure 3:**
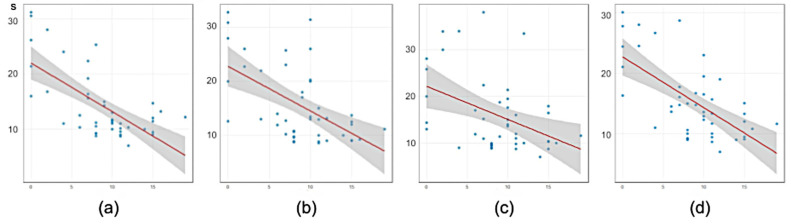
Correlations plots of Leiter-3 Attention Sustained subtest scores (horizontal axis) with the Red Balls test performance time (the vertical axis): (**a**) first subtest, (**b**) second subtest, (**c**) third subtest, and (**d**) averaged over three subtests.

A convergent validity analysis showed that the data of the Leiter-3 Attention Sustained subtest were significantly negatively correlated with mean test performance time (r = −0.66, *p* < 0.001, N = 44) in the Red Balls test. The significance of the correlation for consistently decreased each of the three subtests: r = −0.664, *p* < 0.001, r = −0.580, *p* < 0.001, r = −0.412, *p* < 0.01 respectively (Figure 3).

We conducted a preliminary examination where we divided children into two subgroups according to their performance on the Attention Stability subtest: higher scoring (8–19 points, considered medium and high scoring) and lower scoring (0–7 points, considered below medium and low scoring). The higher scoring subgroup completed the first subtest of the Red Balls test in an average of 11 s (Std = 3.7), while the low scoring group completed it in an average of 20 s (Std = 6.8). We can therefore propose that if a child takes more than 15 s to complete one subtest of the Red Balls test, they are likely to score below medium or worse on the Leiter-3 attention subtest.

### 3.3. Test–Retest Reliability

Repeated testing showed a significant correlation between the number of trials completed on day 1 and day 2 in the first subtest of the White Dots test (r = 0.72, *p* < 0.001, N = 34) and in the Red Balls test (r = 0.82, *p* < 0.001, N = 27), which is in line with previous studies indicating good reliability [19].

### 3.4. Qualitative Assessment of Test Performance

On the basis of analyzing the video recordings of task performance, the following additional criteria for assessment of both tasks were identified:

1. Number of trials performed with verbal stimulation;
2. Number of trials performed when the assessor pointed to the stimulus with a gesture.

These criteria can be used to assess attentional development in cases where the child did not react to stimuli independently.

We also propose additional criteria for assessing children’s task performance that may be potentially useful for characterizing a child’s attention:

1. A child’s tendency to point to a stimulus;
2. A child’s tendency to control gaze with turns of the head;
3. A tendency to ignore a particular area of the screen;
4. Other types of outside assistance used to help a child improve task performance.

## 4. Discussion

The current study has demonstrated that the methods we have developed are effective in assessing voluntary attention in children with neurodevelopmental disorders, although further work is needed to investigate a broader range of disorders. Both tasks correlated significantly with scores on the Attention Sustained subtest of the Leiter International Performance Scale (Leiter-3), indicating the good convergent validity of both tests. Our eye-tracker tests allowed differentiated assessment of voluntary attention in children who received minimal scores on the Attention Sustained subtest, which indicates that our methods can be used to assess the dynamics of voluntary attention development in subthreshold cases for the Leiter-3 subtest. Both tests showed high values of data test–retest reliability.

### 4.1. The White Dots Test

Only 76% of children completed all four subtests of the White Dots test, and both the first and second subtests showed a significant correlation with the performance of the Leiter-3 Attention Sustained subtest. This indicates the redundancy of administering all four subtests. Children aged 3 to 4 years were predominantly represented among those who failed to complete all four subtests. The second subtest was not completed by 13% of children, who were mostly 3–4 years old. When performing the subtest with a stimulus onset asynchrony of 1 s, many 3- to 4-year-olds directed their gaze to the location of the stimulus after it had disappeared. All children who performed well in the 1 s session were older than 5 years 9 months. This suggests that the subtest with 2 s SOA is more suitable for children aged 3–4 and those with more severe neurodevelopmental disorders. On the other hand, the subtest with a 1-second SOA might be more informative for children aged five years and older.

Based on these data, we suggest the following changes to the White Dots test:

1. For children aged 5 and older, the test should consist of subtest 1 and subtest 2 with an SOA of 2 s and 1 s, respectively.
2. For children aged 3–4, and in cases where a child older than 5 years completes the first subtest only with adult assistance, the test should be reduced to subtest 1 with an SOA of 2 s.
3. If the first two subtests are performed well, children aged 5–8 years can be given two additional subtests to further assess neurodynamic performance.

Significant correlations between the Attention Sustained subtest and the White Dots test were found using both video analysis and automatic determination of the number of trials completed. In the former case, however, the correlation was more pronounced. A disadvantage of the automated determination is that it does not take into account the use and type of adult assistance; it shows how many trials were performed in total. Thus, automatic analysis of task performance can be used for the quick screening of attention features, but for a more complete picture of the child’s developmental characteristics of voluntary attention, video analysis is preferable.

Furthermore, the mean latency of fixation on the stimulus in the completed trials was determined for the two subtests of the White Dots test. The correlation between this latency and scores on the Attention Sustained subtest was found to be moderate for the first subtest, but they were not significant for the second subtest. This is in contrast with the high levels of significance observed for the correlation between the Attention Sustained and the number of trials completed. It is noteworthy that in de Jong et al. (2016), the infants’ gaze latency measure was also not significant in the three-factor model of attention [15]. The other measures that were analyzed in the UTATE study [15], such as the number of fixations and re-fixations within the zone of interest, and the accuracy of fixations, are not suitable for our tasks due to their specific design. For instance, the duration of stimulus presentation in the White Dots test influences the number and duration of fixations within the zone of interest. The metrics used in our study are sufficiently informative to provide an assessment of attentional development in children while remaining simple and quick for assessors to administer and analyze.

### 4.2. The Red Balls Test

The Red Balls test was easier for children to complete and required less adult assistance compared to the White Dots test. It was shorter, more colorful, and did not have the gaze marker on the screen that could distract some kids. The mean time taken to complete the subtests significantly correlated with the score of the Attention Sustained scale. The correlations between the time taken to complete each subtest and the score on the Attention Sustained scale also were significant but slightly decreased from the first to the third subtest from high to moderate. It is therefore preferable to use the mean time taken to complete three subtests, but in cases where children showed difficulty completing the first subtest, it is possible to limit the experiment to only the first one. Analyses of changes in subtest performance from the first to the last subtest could provide additional insights into the child’s neurodynamic characteristics such as increased fatigue compared to neurotypical peers.

### 4.3. Verbal and Gestural Assistance

Our methods utilize the help of an adult in case of difficulties in performing the tests. The reasons for this were the following. Preliminary testing of the White Dots test showed that many children with developmental disorders stopped performing the task without adult support. This reduced the informativeness of the test for children with more pronounced developmental disorders, because in this case, children simply received low scores or did not complete the test. At the same time, some children only needed to be reminded of the task once and continued to complete it, while others continued to complete the task only with the help of adult gestures. Thus, children who score poorly on the test without adult assistance may have different levels of voluntary attention, but using adult assistance with the test may help to detect this. The main analysis of the White Dots test is based on counting the number of independently completed tasks. But in the case of very low scores on the main analysis, analyzing the number of tasks completed with the help of an adult will be useful. In the Red Balls test, verbal or gestural assistance was used if children did not look at the stimuli for longer than 3 s; the more help children needed, the longer it took to complete the test. In further studies, it is necessary to develop more detailed criteria for analyzing adult assistance in the context of assessing the level of development of voluntary attention.

### 4.4. Qualitative Analysis of the Tests

For both tests, we additionally developed criteria for the qualitative analysis of test performance, which allow us to take into account the peculiarities of task performance and adult assistance. This allows a more complete representation of the level of development of the child’s voluntary attention, especially in younger children and those with more severe neurodevelopmental disorders. This qualitative analysis supports Luria’s description of the developmental trajectory of voluntary attention in early childhood. According to Luria, attention initially follows the words and gestures of an adult, then moves to the child pointing to objects themselves, and later develops into voluntary attention mediated by internal speech processes [24]. In our study, we observed a similar pattern of attentional development in older children with developmental delays. For example, some children with severe developmental delays required constant verbal and gestural support from adults to complete the tasks. Other children needed only periodic verbal reminders to look at the stimulus. A third group of children could complete tasks independently after a single repetition of the instruction, but they tended to point to the stimulus with their fingers. Interestingly, the quality of task performance decreased when children were unable to point to the stimulus with their finger. As the level of task performance increased, children were able to complete tasks independently without pointing gestures, requiring only occasional reminders from adults. Finally, some children performed all tasks completely without any assistance from adults.

Our proposed tests provide valuable information about a child’s attentional development and allow for a quick and differentiated assessment of attention in children even in cases of a very low score in standard attention tests. The advantage of the White Dots test is that the assessor can see where the child is looking while the child is completing the task. The assessment of the test is very simple and can be completed either while the test is in progress or by video recording of the screen in any convenient way. The advantage of the Red Dots test is that it is simpler and more appealing to children, and it may be able to assess the attention of children with more severe developmental disabilities. However, this test requires more extensive further research to identify performance norms for different age groups. Screen size may also influence the time taken to complete the test, making the test more demanding in terms of monitor technical requirements.

### 4.5. The Tests and Posner Attention Systems

Another discussion point is which aspects of attention our methods assess. A prevalent framework in attention diagnostic methods is Posner’s theory, which identifies three attention systems: orienting, alertness, and executive control [25]. Numerous eye-tracking studies [15,16,17,18] and behavioral tests [7,8,26] attempt to differentiate between these attention systems in children. However, there is still disagreement about which subtests correspond to which attention system. For instance, the Attention Sustained subtest of the Leiter-3 is very similar to the “Selective Attention Test” used in the TEA-Ch and ECAB methods in terms of its tasks and design [7,8,9,26]. Moreover, some researchers have suggested that the three components of attention can only be differentially assessed in children over 4.5 years of age [7,27,28]. The UTATE has shown the feasibility of assessing three attention systems in 18-month-old infants using eye tracking; however, many of the parameters measured overlap between the different attention systems [15]. Lezak et al. (1995) pointed that the clinical manifestations of differences between different attention systems are not so obvious [29]. We believe that our tasks engage all three attentional systems: orienting attention—the child needs to detect a new stimulus in a new location; sustaining attention—the child needs to maintain a certain level of alertness to perform a monotonous task; controlling attention—a child needs to perform a task given by an adult. While our proposed tests cannot assess each of these attention systems separately, they provide valuable information about a child’s development in general.

### 4.6. Limitations and Future Investigations

The technique is not applicable to all children with developmental disabilities; some children in the present study were unable to complete the tasks, refused to stay seated, and refused to look at the screen.

The White Dots test is aimed at assessing voluntary attention in children with developmental disabilities and is of little value for assessing neurotypical children, at least the subtest with an SOA of 2 s. Further research is needed to evaluate the ability of the Red Balls test to assess voluntary attention in typically developing children and to determine age-specific performance norms for the subtests.

In the current study, the tests were tested only for a limited set of development disorders. Further research is needed to explore the application of these techniques in groups of children with other developmental disorders, especially with motor impairments and children with cerebral visual impairments. We propose that our approaches may be suitable for assessing children with gaze control and motor disorders, but some adjustments to the test parameters might be necessary.

A wide age range was used in the present study to assess the feasibility of applying our approach to children of different ages, but further, more extensive research is needed for each age group.

In this work, we did not evaluate how well the tests correlate with intelligence level or speech understanding. We also did not set the task of identifying how children from different clinical subgroups perform differently on these tests, which is the goal of future research.

## 5. Conclusions

Our findings suggest that the tests presented in this study can be used to evaluate voluntary attention in children with developmental disorders. The tests demonstrated good convergent validity and test–retest reliability. They can also help track the development of voluntary attention even in children with low scores on the Attention Sustained subtest or in cases where standardized tests cannot be administered.

These findings suggest that further research on the use of these tests in children with various developmental disorders, particularly in those unable to perform standardized attention tests, is warranted.

## Figures and Tables

**Table 1:** Distribution of 80 children by age, sex, and diagnosis.

	Total	3 y.o.	4 y.o.	5 y.o.	6 y.o.	7–8. y.o.
Number of children	80	8	19	18	18	17
Number of males	55	4	16	11	10	14
PDD	23	2	6	6	4	5
ELD	24	2	9	4	5	4
AD	8	0	2	3	2	1
Other diagnosis ^1^	25	3	3	5	7	7

^1^ Mild mental retardation, attention deficit hyperactivity disorder, other and unspecified speech disturbances, spastic diplegic cerebral palsy, encephalopathy, unspecified, other disorders of the nervous system. PDD—other pervasive developmental disorders; ELD—expressive language disorder; AD—autistic disorder.

**Table 2:** The distribution of children (N = 72) by level of performance on the Attention Sustained subtest and the average number of independently completed trials and its standard deviation for each subgroup for subtest 1 and subtest 2 of the White Dots test.

Groups by Attention Sustained Subtest	0–3 Very Low	4–7 Low and Below Medium	8–12 Medium	13–19 Above Medium and High
N	13	14	31	13
Number completed trials in subtest 1	10.36 (Std 7.8)	23.14 (Std 5.12)	25.74 (Std 2.92)	27 (Std 2.7)
Number completed trials in subtest 2	5.2 (Std 4.13)	17.58 (Std 6.33)	21.53 (Std 6.05)	24.42 (Std 3.08)

## Data Availability

The data presented in this study are available on request from the corresponding author.
